# A distal convoluted tubule‐specific isoform of murine SLC41A3 extrudes magnesium

**DOI:** 10.1111/apha.70018

**Published:** 2025-02-11

**Authors:** Gijs A. C. Franken, Willem Bosman, Hyun Jun Jung, Caro Bos, Femke Latta, Mark Knepper, Joost G. J. Hoenderop, Jeroen H. F. de Baaij

**Affiliations:** ^1^ Department of Medical BioSciences Radboud University Medical Center Nijmegen The Netherlands; ^2^ Division of Nephrology, Department of Medicine Johns Hopkins University School of Medicine Baltimore Maryland USA; ^3^ Epithelial Systems Biology Laboratory, Systems Biology Center National Heart, Lung, and Blood Institute, National Institutes of Health Bethesda Maryland USA

**Keywords:** alternative transcript, kidney, magnesium, physiology

## Abstract

**Background:**

The distal convoluted tubule (DCT) plays an indispensable role in magnesium (Mg^2+^) reabsorption in the kidney. Yet, the extrusion mechanism of Mg^2+^ has not been identified. The solute carrier 41A3 (SLC41A3) has been suggested to be involved in Mg^2+^ extrusion, but this has never been conclusively demonstrated.

**Methods:**

Using available RNA‐sequencing data and real‐time quantitative PCR, expression of two alternative *Slc41a3* transcripts, encoding isoform (Iso) 1 or 2, were assessed in kidney and isolated DCT tubules. HEK293 or HAP1 cells were transfected with plasmids expressing either of the isoforms, followed by ^25^Mg^2+^ transport studies. Identification of cis‐regulatory elements (CRE) was achieved by combining data from publicly available ATAC sequencing data and luciferase assays.

**Results:**

Gene expression studies revealed a distinct transcript of *Slc41a3* in the DCT with an alternative promoter, leading to a protein with a unique N‐terminus; SLC41A3‐Iso 2. HEK293 cells overexpressing SLC41A3‐Iso 2, but not ‐Iso 1, exhibited 2.7‐fold and 1.6‐fold higher ^25^Mg^2+^ uptake and extrusion, compared to mock, respectively. The transport was independent of Na^+^, of the Mg^2+^ channel TRPM7 or of transporters CNNM3 and ‐4. We identified a CRE accessible in the DCT, ±2.8kb upstream of the transcript. The presence of the CRE increased the *Slc41a3‐Iso 2* promoter activity 3.8‐fold following luciferase assays, indicating the CRE contains an enhancer function.

**Conclusion:**

In conclusion, we identified two alternative transcripts of *Slc41a3* in mouse. *Slc41a3‐Iso 2* is enriched within the DCT using specific gene regulatory elements. We speculate that specifically in the DCT, SLC41A3‐Iso 2 orchestrates Mg^2+^ extrusion.

## 1 INTRODUCTION

Renal magnesium (Mg^2+^) reabsorption is the main regulator of systemic Mg^2+^ homeostasis. Disturbances in renal Mg^2+^ handling lead to hypomagnesaemia (serum Mg^2+^ levels <0.7 mmol/L) and as a result to fatigue, muscle cramps, seizures, and cardiac arrhythmias.^1^ Within the kidney, Mg^2+^ is mainly reabsorbed from the pro‐urine in a paracellular fashion in the proximal tubule and the thick ascending limb of the loop of Henle (TAL). The distal convoluted tubule (DCT) reabsorbs approximately 10% of filtered Mg^2+^ in a transcellular way.^2^ Transient receptor potential melastatin (TRPM) type 6 and −7 channels are responsible for luminal Mg^2+^ influx in the DCT.^3^, ^4^, ^5^ The subsequent Mg^2+^ extrusion mechanism currently remains elusive. Multiple proteins have been proposed as the Mg^2+^ extruder in the DCT, including Cyclin M2 (CNNM2) and solute carrier family 41 (SLC41) members A1 and A3.^6^, ^7^, ^8^

The SLC41 protein family consists of members A1‐3, which all contain the highly conserved, prokaryotic Magnesium Transporter E (MgtE) domain.^9^, ^10^, ^11^ Of the three members, SLC41A3 is highly expressed in the DCT and its transcript is upregulated upon low Mg^2+^ intake in mice.^12^ Mice deficient in SLC41A3 (*Slc41a3* ^*−/−*^) display hypomagnesaemia and increased expression of magnesiotropic genes, such as *Trpm6* and *Slc41a1*.^7^ Initially, SLC41A3 was predicted to be a plasma membrane Na^+^/Mg^2+^ exchanger.^8^, ^13^ However, characterization of SLC41A3‐mediated Mg^2+^ transport suggests that this protein primarily localizes to the mitochondria, despite the absence of a mitochondrial localization sequence.^14^ Isolated mitochondria of HEK293 cells overexpressing SLC41A3 exhibited increased Na^+^‐dependent Mg^2+^ efflux, as demonstrated in studies using the Mg^2+^‐sensitive probe Mag‐Fura‐2.^14^ The link between a Na^+^/Mg^2+^ mitochondrial efflux transporter and systemic Mg^2+^ homeostasis remains obscure.

In this study, the renal expression of an alternative SLC41A3 transcript with a distinct N‐terminus (SLC41A3‐Iso 2) was investigated. Subsequently, the function of murine SLC41A3 isoforms was studied using ^25^Mg^2+^ transport assays, to identify their role in renal Mg^2+^ extrusion. Subcellular expression was investigated using confocal microscopy and cell surface biotinylation. Lastly, the transcriptional regulation of SLC41A3‐Iso 2 was explored by publicly available ATAC‐sequencing data and luciferase assays.

## 2 RESULTS

### 2.1 Enriched expression of *Slc41a3 isoform 2* in the distal convoluted tubule

**FIGURE 1:**
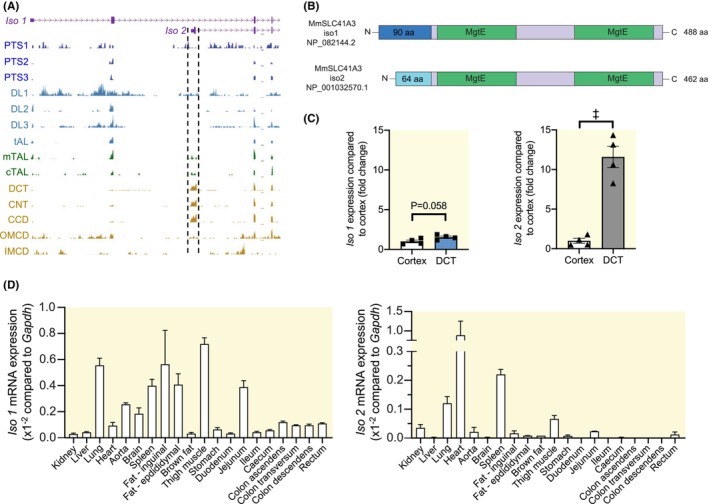
Isoform‐specific expression pattern of mouse *Slc41a3*. (A) *Slc41a3* exon 1–3 RNA‐seq data of different nephron segments.^15^ The region flanking the *Slc41a3 isoform 2* TSS is marked with dotted lines. (B) Schematic overview of the shared domains between murine SLC41A3 isoforms. The colors correspond between isoforms when high amino acid identity was found, using MUSCLE for the alignment.^16^ (C) RT‐qPCR data of *Slc41a3* isoform expression in the DCT compared to the whole kidney cortex. Data points represent four independent experiments. Data are presented as mean ± SEM. ^‡^ *p* < 0.001. (D) RT‐qPCR data of *Slc41a3* isoform expression in different mouse tissues from three male C57BL/6 mice. CCD, cortical collecting duct; CNT, connecting tubule; cTAL, cortical thick ascending limb of Henle's loop; DCT, distal convoluted tubule; DL1‐3, descending limb of Henle's loop segments 1–3; IMCD, inner medullary collecting duct; Iso 1/2, mouse SLC41A3 isoform 1/2; mTAL, medullary thick ascending limb of Henle's loop; OMCD, outer medullary collecting duct; PTS1‐3, proximal tubule segments 1–3; tAL, thin ascending limb of Henle's loop.

Analysis of the *Slc41a3* gene in RNA‐sequencing data of micro‐dissected tubules from mouse kidney^15^ demonstrated specific expression of *Slc41a3 isoform 2* (*Slc41a3‐Iso 2*; NM_001037493.2, NP_001032570.1) in distal tubules (distal convoluted tubule [DCT]), connecting tubule (CNT), and cortical collecting duct (CCD). (Figure 1A). *Slc41a3‐Iso 2* makes use of an alternative transcription start site (TSS) in the second intron of the *Slc41a3 isoform 1* gene (*Slc41a3‐Iso 1*; NM_027868.2, NP_082144.2). This gives rise to isoforms SLC41A3‐Iso 1 and ‐Iso 2 proteins with their own distinct N‐termini of different sizes (90 and 64 amino acids, respectively) (Figure 1B).

To confirm enriched gene expression of *Slc41a3‐Iso 2* in the DCT, mRNA expression of both isoforms was assessed in isolated DCT tubules and the kidney cortex. Expression of *Slc41a3‐Iso 2* was 11.6‐fold higher in the DCT compared to the cortex (*p* = 0.0003), while *Slc41a3‐Iso 1* expression was not significantly different between the two tissues (1.5‐fold higher in DCT, *p* = 0.058) (Figure 1C). We also assessed expression of *Slc41a3* isoforms in other organ systems using mRNA isolated from different mouse tissues. *Slc41a3‐Iso 1* was ubiquitously expressed with highest expression in muscle, lung, and fat tissues, while *Slc41a3‐Iso 2* showed a more restricted expression pattern and a strong enrichment in the heart (Figure 1D).

### 2.2 Mouse SLC41A3 isoforms have specific effects on Mg^2+^ transport

**FIGURE 2:**
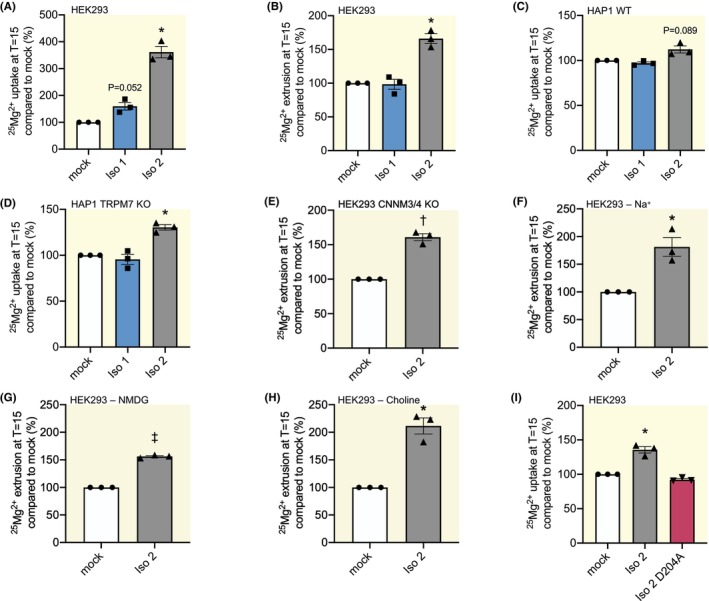
Isoform‐specific effects on SLC41A3‐mediated Mg^2+^ transport. (A, B) ^25^Mg^2+^ uptake (A) and extrusion (B) in HEK293 cells transfected with mouse SLC41A3 isoforms compared to mock‐transfected cells. (C, D) ^25^Mg^2+^ uptake levels in HAP1 WT (C) or HAP1 TRPM7 KO (D) cells transfected with mock or mouse SLC41A3 isoforms. (E) ^25^Mg^2+^ extrusion levels in mock‐ or mouse SLC41A3‐transfected CNNM3/CNNM4 double knock‐out HEK293 cells. (F–H) ^25^Mg^2+^ extrusion in mock or mouse SLC41A3‐Iso 2‐transfected HEK293 cells in the presence of Na^+^ (F), NMDG (G) or choline (H) to assess Na^+^‐dependency. (I) ^25^Mg^2+^ uptake in HEK293 cells transfected with mouse SLC41A3 isoform 2 WT or the p.D204A variant compared to mock‐transfected cells. Data points represent independent experiments. Data are presented as mean ± SEM. *p < 0.025 (A–D, I) or <0.05 (E–H), ^†^ *p* < 0.01, ^‡^ *p* < 0.001. Iso 1/2, mouse SLC41A3 isoform 1/2; NMDG, *N*‐Methyl‐D‐glucamine.

To determine the isoform‐specific effects of SLC41A3 on Mg^2+^ transport, HEK293 cells were transfected with constructs encoding SLC41A3‐Iso 1 or ‐Iso 2 and subjected to transport assays with the stable isotope ^25^Mg^2+^. This isotope allows to establish the direction of Mg^2+^ transport over the cell membrane, but does not reflect the total intracellular Mg^2+^ concentrations. To determine Mg^2+^ influx, cells were exposed to extracellular ^25^Mg^2+^ for 15 minutes. Cells transfected with SLC41A3‐Iso 2 showed a 3.6‐fold higher uptake of ^25^Mg^2+^ than cells transfected with empty vector (mock) (*p* = 0.0065) (Figure 2A). SLC41A3‐Iso 1 did not induce a significant increase in ^25^Mg^2+^ uptake compared to control (1.6‐fold, *p* = 0.052) in this time span. However, both SCL41A3‐Iso 1 and ‐Iso 2‐transfected cells showed an increase of relative intracellular ^25^Mg^2+^ content compared to mock, relative to total natural Mg^2+^ (^26^Mg and ^24^Mg) after 24 h of exposure to ^25^Mg^2+^ (74.4% and 79.1% vs. 67.5%, *p* = 0.035 and 0.0034, respectively) (Figure S2A). This suggests both isoforms increase Mg^2+^ influx, albeit at different velocity. To determine if the SLC41A3 isoforms also induced Mg^2+^ extrusion, ^25^Mg^2+^ extrusion experiments were performed. Following ^25^Mg^2+^ loading overnight, SLC41A3‐Iso 2‐transfected cells also showed increased ^25^Mg^2+^ extrusion (1.7‐fold compared to mock, *p* = 0.012), while extrusion levels of SLC41A3‐Iso 1‐transfected cells were comparable to controls (98%) (Figure 2B). For the extrusion experiments, cells were loaded for 48 h with ^25^Mg^2+^ and intracellular levels at the start of the experiments were comparable in all conditions (Figure S2B). Despite its higher functional activity, protein expression level of SLC41A3‐Iso 2 during the uptake and extrusion experiments was much lower than for SLC41A3‐Iso 1 (Figure S2C,D).

To assess whether the observed effects were dependent on other Mg^2+^ transporters or channels, the uptake assay was repeated in TRPM7 KO cells. The Mg^2+^ channel TRPM7 is considered the main entry for Mg^2+^ into cells.^17^ We used KBM‐7 chronic myelogenous leukemia‐derived HAP1 cell lines, which are haploid for most genes and therefore easily genetically manipulated. In addition, these TRPM7 KO cells have been used in multiple studies to investigate TRPM7 function.^18^, ^19^ In the absence of TRPM7, SLC41A3‐Iso 2, but not SLC41A3‐Iso 1, still increases ^25^Mg^2+^ uptake (1.3‐fold compared to mock, *p* = 0.0092) (Figure 2D). In HAP1 WT cells, this increase was not observed, but it should be noted that transfection efficiency was higher in the TPRM7 KO HAP1 cells (Figure 2C,D, Figure S2E). In addition, we assessed whether the extrusion effect of SLC41A3‐Iso 2 was mediated by CNNM3 and CNNM4, known players in Mg^2+^ extrusion.^18^ To investigate this hypothesis, CNNM3 and − 4 double‐knock‐out HEK293 cells were used. SLC41A3‐Iso 2‐expressing cells showed higher Mg^2+^ extrusion in CNNM3 and CNNM4 double‐knock‐out HEK293 cells compared to mock (1.6‐fold, *p* = 0.0068) (Figure 2E).

To test the hypothesis that SLC41A3‐iso 2 is dependent on Na^+^ for Mg^2+^ extrusion, *N*‐Methyl‐D‐glucamine (NMDG) and choline were used to substitute Na^+^ whilst maintaining an equal osmolarity to the original buffer. Overexpression of SLC41A3‐iso 2 resulted in an increased Mg^2+^ extrusion in all conditions (1.8‐fold (*p* = 0.040), 1.6‐fold (*p* = 0.0007), and 2.1‐fold (*p* = 0.017) compared to mock for Na^+^, NMDG and choline, respectively) (Figure 2F–H).

Lastly, we introduced the p.D204A variant in SLC41A3‐Iso 2, which affects a conserved motif in SLC41A3 that is thought to form the Mg^2+^‐conducting pore based on homology with the prokaryotic Mg^2+^ transporter MgtE.^9^ This allows us to determine whether Mg^2+^ extrusion is mediated directly via SLC41A3 or mediated via another mechanism. Indeed, ^25^Mg^2+^ uptake levels were similar to mock‐transfected cells when SLC41A3‐Iso 2‐p.D204A was transfected (Figure 2I).

### 2.3 Mouse SLC41A3 isoforms have a partial overlapping but distinct localization pattern

**FIGURE 3:**
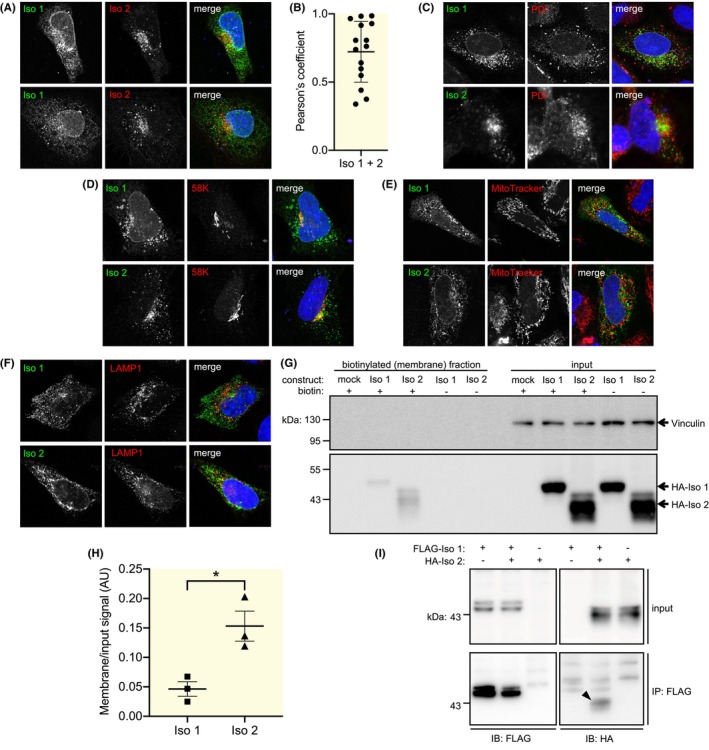
Isoform‐specific subcellular localization patterns of mouse SLC41A3 isoforms. (A) Representative co‐immunostainings of mouse SLC41A3 isoforms transfected in HeLa cells. (B) Quantification of colocalization of mouse SLC41A3 isoforms. Data points represent individual cells. Data are presented as mean ± SD. (C–F) Representative co‐immunostainings of mouse SLC41A3 isoforms and PDI (marker of the endoplasmatic reticulum) (C), 58 K (marker of theGolgi apparatus) (D), MitoTracker (staining of mitochondria) (E) and LAMP1 (marker of the lysosomes) (F) in HeLa cells. (G) Representative Western blot of the cell surface biotinylation assay. (H) Quantification of the cell surface biotinylation assay. Data points represent independent experiments. Data are presented as mean ± SEM. *p < 0.05 (I) Co‐immunoprecipitation of mouse SLC41A3 isoforms. The bait protein was FLAG‐tagged isoform 1, the target protein was HA‐tagged isoform 2 (indicated with arrowhead). IB, immunoblotting; Iso 1/2, mouse SLC41A3 isoform 1/2; IP, immunoprecipitation.

To assess whether SLC41A3 isoforms have different subcellular localizations, we used HeLa cells as they have larger cell bodies compared to HEK293 cells, making the assessment of subcellular structures easier. HeLa cells were co‐transfected with both isoforms making use of constructs encoding each isoform with a different tag. FLAG‐SLC41A3‐Iso 1 and HA‐SLC41A3‐Iso 2 both showed a dot‐like expression pattern throughout the cell (Figure 3A). In dense regions close to the nucleus colocalization of the isoforms is observed, while they show distinct localization in the rest of the cell, resulting in an average Pearson's coefficient of 0.72 (±0.22) (Figure 3B). Next, SLC41A3 was stained together with markers for different organelles (Figure 3C–F). While some localization was observed in the endoplasmatic reticulum, Golgi apparatus, and lysosomes, these stainings showed that neither SLC41A3 isoform is limited to one of these cellular compartments. Similarly, there was no strong colocalization with the mitochondria (Figure 3E). To determine expression at the cell membrane, we performed a cell surface biotinylation assay. Both isoforms are present on the membrane, but the relative membrane expression compared to total expression was higher for SLC41A3‐Iso 2 versus SLC41A3‐Iso 1 (0.15 versus 0.046, *p* = 0.020) (Figure 3G,H). Lastly, since the co‐stainings showed a partial overlap and proteins of the SLC41 family may act as dimers, we assessed whether the isoforms can interact with each other. HEK293 cells were co‐transfected with both isoforms and used for co‐immunoprecipitation, which revealed that HA‐SLC41A3‐Iso 2 is present in the pulldown when FLAG‐SLC41A3‐Iso 1 is precipitated (Figure 3I).

### 2.4 Transcriptional regulation of *Slc41a3 isoform 2* by a cis‐regulatory element

**FIGURE 4:**
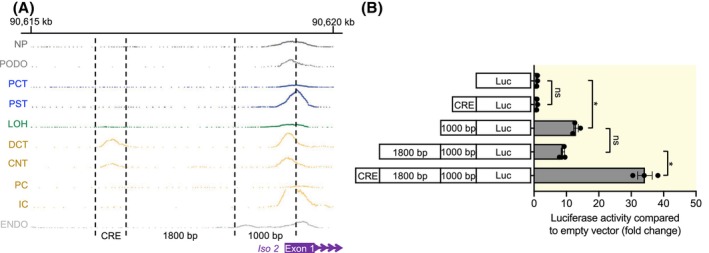
A distal convoluted tubule‐specific open DNA region upstream of *Slc41a3 isoform 2* can regulate downstream expression. (A) snATAC‐seq data of different mouse nephron segments.^17^ Relevant DNA regions upstream of the *Slc41a3 isoform 2* TSS are marked with dotted lines. (B) Luciferase assay of the different regions shown in A cloned in front of the luciferase gene. Data are obtained from three independent experiments and are presented as mean ± SEM. *p < 0.05. CNT, connecting tubule; DCT, distal convoluted tubule; ENDO, endothelium; IC, intercalated cells of collecting duct; *Iso 2*, mouse *Slc41a3 isoform 2*; LOH, loop of henle; NP, nephron progenitors; PC, principal cells of collecting duct; PCT, proximal convoluted tubule; PODO, podocytes; PST, proximal straight tubule.

To find an explanation for the segment‐specific expression of SLC41A3 isoforms, publicly available single‐nucleus Assay for Transposase‐Accessible Chromatin‐sequencing (snATAC‐seq) data of murine nephron cells were used.^20^ An open 477 bp DNA region (cis‐regulatory element, CRE) was found ~2800 bp upstream of the *Slc41a3‐Iso 2* TSS, which was open only in the DCT and CNT (Figure 4A). A luciferase assay was subsequently used to assess whether this CRE regulates *Slc41a3‐Iso 2* transcription. By itself, the CRE did not increase luciferase activity compared to the control vector while the putative *Slc41a3‐Iso 2* promoter (1000 bp upstream of the TSS) increased the activity by 13.0‐fold (*p* = 0.014) (Figure 4B). Addition of the 1800 bp until the CRE did not further increase transcriptional activity, while the addition of the CRE resulted in a 3.8‐fold increase compared to the promoter and 1800 bp region alone (*p* = 0.031) (Figure 4B).

### 2.5 Less distinct isoform‐specific effects of human SLC41A3

**FIGURE 5:**
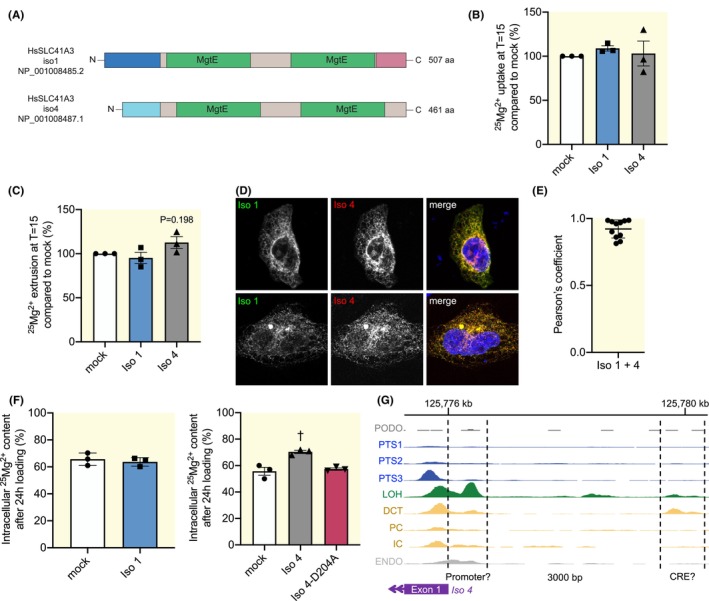
Function and localization of human SLC41A3 isoforms 1 and 4. (A) Schematic overview of the shared domains between human SLC41A3 isoforms. The colors correspond between isoforms when high amino acid identity was found, using MUSCLE for the alignment.^16^ (B, C) ^25^Mg^2+^ uptake (B) and extrusion (C) in HEK293 cells transfected with human SLC41A3 isoforms compared to mock‐transfected cells. (D) Representative co‐immunostainings of human SLC41A3 isoforms transfected in HeLa cells. (E) Quantification of colocalization of human SLC41A3 isoforms. (F) Intracellular ^25^Mg^2+^ content in cells transfected with mock or human SLC41A3 isoform 1, 4 or 4‐p.D204A and loaded with 1 mmol/L ^25^MgO for 24 h. (G) snATAC‐seq data of different human nephron segments.^19^ Relevant DNA regions upstream of the *SLC41A3 isoform 4* TSS are marked with dotted lines. Data points represent three independent experiments (B, C, F) or individual cells (E). Data are presented as mean ± SEM (B, C, F) or ±SD (E). ^†^ *p* < 0.01. DCT, distal convoluted tubule; ENDO, endothelium; IC, intercalated cells of collecting duct; Iso 1/4, human SLC41A3 isoform 1/4; LOH, loop of Henle; PC, principal cells of collecting duct; PODO, podocytes; PTS1‐3, proximal tubule segments 1–3.

To examine the relevance of our findings for human physiology, the presence of SLC41A3 isoforms in the human transcriptome was examined using BLAST. We identified homologues of both isoforms. Human *SLC41A3 isoform 4* (*hSLC41A3‐Iso 4*; NM_001008487.2, NP_001008487.1) also uses an alternative TSS in intron 2 of *SLC41A3 isoform 1* (*hSLC41A3‐Iso 1*; NM_001008485.2, NP_001008485.2; Figure 5A, Figure S1). Of note, hSLC41A3‐Iso 1 has a C‐terminus distinct from all other isoforms.

Based on the homology between SLC41A3‐Iso 2 and hSLC41A3‐Iso 4, we assessed whether hSLC41A3‐Iso 1 and ‐Iso 4 show the same isoform‐specific effects as mouse SLC41A3‐Iso 1 and ‐Iso 2. Both human isoforms did not affect ^25^Mg^2+^ uptake compared to mock‐transfected cells (Figure 5B). Similarly, although transfection of hSLC41A3‐Iso 4 seemed to slightly increase ^25^Mg^2+^ extrusion, there were no significant effects on extrusion (Figure 5C). Protein expression levels of hSLC41A3‐Iso 1 and ‐Iso 4 were similar in these experiments (Figure S2F). Co‐stainings of HA‐hSLC41A3‐Iso 1 and FLAG‐hSLC41A3‐Iso 4 revealed an almost perfect colocalization in all cells (Pearson's coefficient of 0.92 (±0.067)) (Figure 5D,E). The only effect of mouse SLC41A3‐Iso 2 reproduced by hSLC41A3‐Iso 4, but not by hSLC41A3‐Iso 1, was an increase in intracellular ^25^Mg^2+^ relative to total Mg^2+^ after 24 h of loading (70.3% vs. 55.7% in mock cells, *p* = 0.0030), which was prevented by introducing the same presumed pore‐affecting variant p.D204A in hSLC41A3‐Iso 4 (Figure 5F). Moreover, human snATAC‐seq data^21^ also reveals an open DNA region upstream of the *SLC41A3‐Iso 4* TSS, which is specifically accessible in the DCT and loop of Henle (Figure 5G). The in silico tool JASPAR was used to identify transcription factors predicted to bind both the mouse and human CREs, which resulted in five candidates (*Esr2, Foxj3, Kf1, Rara* and *Znf470*) (Table S2). All five candidates show low expression in the mouse DCT based on RNA‐seq data.^15^

## 3 DISCUSSION

The largest unresolved mechanism in renal Mg^2+^ transport is the molecular identity of the putative basolateral Na^+^/Mg^2+^ exchanger in the DCT. Here, we propose that an alternative isoform of the *Slc41a3* gene could contribute to Mg^2+^ extrusion. This conclusion is based on the following findings: (*i*) *Slc41a3‐Iso 2* expression is enriched in the DCT; (*ii*) SLC41A3 isoforms facilitate Mg^2+^ transport and SLC41A3‐Iso 2 increases Mg^2+^ extrusion; (*iii*) *Slc41a3‐Iso 2* expression is regulated by a cis‐regulatory element (CRE), accessible in the DCT; and (*iv*) the presence of this shorter isoform is conserved in humans. This study is, to our knowledge, the first to describe the presence and function of different SLC41A3 isoforms in the kidney.

The existence of isoform‐specific effects has important implications for the understanding of SLC41A3 function. Deletion of *Slc41a3* in mice resulted in lowered serum Mg^2+^ levels and increased renal urinary Mg^2+^ wasting.^7^ This suggests that SLC41A3 regulates Mg^2+^ homeostasis, either directly or indirectly. Here, we have shown that only SLC41A3‐Iso 2 can act as a bidirectional Mg^2+^ transporter on the cell membrane based on increased ^25^Mg^2+^ uptake and extrusion. This bidirectional Mg^2+^ flux has also been observed for SLC41A1 and SLC41A2^8^, ^13^ and could be explained by the fact that the ^25^Mg^2+^ assay does not reflect intracellular Mg^2+^ concentrations, meaning ^25^Mg^2+^ uptake can still be observed in the context of Mg^2+^ extrusion. It has been postulated that members of the SLC41 protein family are Na^+^/Mg^2+^ exchangers,^8^, ^13^, ^14^ though we could not confirm this hypothesis. Replacement of Na^+^ with NMDG also does not affect SLC41A1‐induced ^25^Mg^2+^ extrusion,^8^ but does abolish SLC41A1‐dependent decreases in intracellular Mg^2+^ concentrations,^22^ indicating Na^+^‐dependency experiments are sensitive to specific conditions and read‐outs. In a physiological setting, Mg^2+^ extrusion requires secondary active transport, which still makes Na^+^ the most likely candidate. Electrophysical studies, such as two‐electrode voltage clamping, could aid in the identification of the counter‐ion.

The lack of detection of Mg^2+^ fluxes by SLC41A3‐Iso 1 is in accordance with Mastrototaro et al., as they demonstrated that Iso 1 solely acts as mitochondrial Mg^2+^‐extruder.^14^ It should be mentioned that human SLC41A3‐Iso 1 function was assessed in this study. Interestingly, using two‐electrode voltage clamping and the Mg^2+^‐sensitive probe MagFura‐2, Mg^2+^‐evoked currents and changes in intracellular Mg^2+^ concentrations were observed in *Xenopus* oocytes overexpressing SLC41A3.^23^ However, this study did not specify which *Slc41a3* isoform cRNA was injected into the oocytes. Furthermore, changes in mitochondrial Mg^2+^‐transport could also affect cellular Mg^2+^ concentrations and currents measured on the cell membrane, as observed for the mitochondrial Mg^2+^ channel Mrs2.^24^, ^25^

The isoform‐specific Mg^2+^ transport function of SLC41A3‐Iso 2 was abolished by mutating the MgtE region which contains the pore, highlighting direct transport by SLC41A3 itself. Indeed, the introduction of this same variant in the homologous residues of MgtE (WP_011228410.1:p.D432A) and SLC41A1 (NP_776253.3:p.D263A) also abolished their Mg^2+^ transport capacity.^8^, ^26^, ^27^ Our findings that SLC41A3‐Iso 2 is a Mg^2+^‐transporter per se were further demonstrated by the fact that it was able to increase influx independent of the Mg^2+^ channel TRPM7. Similarly, Mg^2+^ influx could be observed by SLC41A2 in TRPM7 deficient DT40 cells.^13^ Moreover, blocking TRPM7 using the non‐selective inhibitor 2‐APB was not sufficient to affect SLC41A1‐mediated Mg^2+^ influx.^8^ Furthermore, deletion of CNNM3 and − 4, proteins known to extrude cellular Mg^2+^, were likewise unable to inhibit SLC41A3‐mediated Mg^2+^ efflux. This together suggests that SLC41A3‐Iso 2, similarly to other SLC41 family members, is an independent, potent Mg^2+^ transporter.

Of note, the potency of SLC41A3‐Iso 2 might have been underestimated in this study, particularly compared to SLC41A3‐Iso 1. Remarkedly, SLC41A3‐Iso 2 protein expression was approximately 35‐fold lower than SLC41A3‐Iso 1, though the same amount of plasmid was used for our studies, with the same backbone. The low expression of a Mg^2+^ extruding protein is in line with previous findings, overexpressing CNNM2 or − 4, which increase Mg^2+^ efflux and decrease intracellular Mg^2+^ concentrations, are expressed to a lower extent compared to their counterparts CNNM1 and ‐3.^18^, ^28^ Translation efficiency, post‐translational modifications, or protein degradation could play a role. For instance, ribosomes are dependent on sufficient intracellular Mg^2+^ concentrations, so increased Mg^2+^ efflux could affect translation efficiency.^29^

In order to fully elucidate the isoform‐specific functions of SLC41A3, it is important to confirm the subcellular localization of the isoforms. Generation of custom‐made antibodies specific for iso 2, based on the unique N‐terminus peptide sequence, was not successful and therefore subcellular localization in vivo could not be addressed (data not shown). Unfortunately, there are no DCT cell lines are available that polarize, which is a known limitation in the field.^30^ We performed biotinylation assays of mouse SLC41A3 in HEK293 cells which showed presence at the cell surface and the relative membrane expression was higher for SLC41A3‐Iso 2 than for ‐Iso 1. Mastrototaro et al. found predominant expression in the mitochondria, but focused on human SLC41A3‐Iso 1.^14^ Our data indicate that the mitochondria as well as the endoplasmatic reticulum, Golgi apparatus and lysosomes are not the main site of mouse SLC41A3 expression. The N‐terminus of SLC41A3 is different in SLC41A3‐Iso 1 and ‐Iso 2, but in silico prediction did not reveal the presence of signal peptides in SLC41A3 isoforms. This suggests that the proteins might not be differentially trafficked or require adaptor proteins that may be cell‐specific.

Next to murine SLC41A3, we also investigated human SLC41A3 isoforms. We found hSLC41A3‐Iso 1 and ‐Iso 4 to be the most similar to murine SLC41A3‐Iso1 and ‐Iso 2 on amino acid identity, respectively. We could not demonstrate distinct effects in our ^25^Mg^2+^ transport assays when overexpressing human SLC41A3 isoforms. However, we showed that, similar to murine SLC41A3‐Iso 2, human SLC41A3‐Iso 4 expressing cells had increased ^25^Mg^2+^ loading, which was abolished by expressing the MgtE‐motif mutant hSLC41A3‐Iso 4‐p.D204A. This proposes that hSLC41A3‐Iso 4 can directly transport Mg^2+^ across the cell membrane. However, the fact there are in total nine isoforms listed on Uniprot underlines that the differences in function of SLC41A3 might be more nuanced in humans. Non‐redundant functional differences might still be present in the other isoforms.

Publicly available RNA‐seq data from tubules micro‐dissected from the kidney demonstrates high expression of *Slc41a3‐Iso 2* in the DCT. We confirmed these findings by isolating DCT cells from the cortex and observed enrichment of *Slc41a3‐Iso 2* in the DCT, in contrast to *Slc41a3‐Iso 1*. An earlier study found that *Slc41a3* transcripts were enriched in the DCT, but did not distinguish between the isoforms.^7^ Genetic inactivation of the *Slc41a3* gene, targeting both alternative transcripts, resulted in hypomagnesaemia.^7^ We hypothesize that particularly *Slc41a3‐Iso 2* contributes to the hypomagnesaemia observed in *Slc41a3* ^*−/−*^ mice, due to disrupted DCT‐mediated Mg^2+^ transport. Interestingly, *Slc41a3* ^−/−^ *and Slc41a1‐Slc41a3* double KO mice both exhibit similar levels of renal Mg^2+^ wasting and decreased serum Mg^2+^ levels, suggesting that SLC41A1 is not involved in renal Mg^2+^ handling.^7^, ^31^ Isoform‐specific knock‐out mice should be generated to determine the contribution of SLC41A3 isoforms in Mg^2+^ homeostasis.

To determine what drives the expression of distinct isoforms in the DCT, we used publicly available snATAC‐seq data to detect regulatory DNA regions in close proximity of the TSS of the *Slc41a3‐Iso 2* gene. Our luciferase data indicate that the identified CRE ~2800 bp upstream of the *Slc41a3‐Iso 2* TSS might be a transcriptionally active region that regulates the distal nephron‐specific expression of *Slc41a3‐Iso 2*. Epigenetic data on active enhancer regions could confirm this, but this is currently unavailable for the different nephron segments. Strikingly, a similar region is observed upstream of *SLC41A3‐Iso 4* in snATAC‐seq data of human DCT cells. Although the mouse and human CREs are not homologous, transcription factors typically only require short motifs that can be present in both. There are other examples of proteins with isoform‐specific expression in the DCT. Lysine deficient protein kinase 1 (WNK1) has a kidney‐specific isoform that is most prominently expressed in the DCT.^32^, ^33^ In addition, the γ‐subunit of the Na^+^/K^+^‐ATPase (FXYD2) has two isoforms with distinct N‐termini, of which isoform b localizes mainly to the DCT and CNT, similar to what has been observed for SLC41A3 in this study.^34^ Lastly, an holistic approach following RNA‐sequencing on micro‐dissected tubules has detected more alternative exon usage along the nephron.^15^ It might be interesting to investigate whether the transcriptional regulatory mechanisms underlying isoform‐specific expression of these different genes are overlapping.

From a physiological point of view, our study adds significant insights to Mg^2+^ extrusion mechanism in the DCT. Our data suggests that SLC41A3‐Iso 2 is an important contributor to Mg^2+^ extrusion. However, our findings do not exclude that other Mg^2+^ extrusion mechanisms in the DCT exist. CNNM2 has been hypothesized to be an alternative to SLC41A3 for Mg^2+^ extrusion in the DCT.^6^, ^35^, ^36^ Indeed, both *Cnnm2* ^+/−^ and kidney‐specific *Cnnm2* ^−/−^ mice demonstrated hypomagnesaemia and renal Mg^2+^‐wasting.^37^, ^38^ Studying how SLC41A3 function and CNNM2 function are interdependent would be highly valuable to the field. However, the absence of reliable DCT cell models currently is a major limitation.^30^

In conclusion, this study provides evidence that SLC41A3 isoform 2 might be the facilitator of Mg^2+^ extrusion in the mouse DCT, based on its enriched expression in the DCT and substantial efflux function. Future studies on cell‐specific isoforms of SLC41A3 and other transport proteins in DCT are essential to elucidate DCT physiology.

## 4 MATERIALS AND METHODS

### 4.1 Reverse transcription quantitative PCR

To assess *Slc41a3* isoform expression in mouse tissues, cDNA generated from RNA isolated from different tissues of three male C57BL/6 mice was used, as described previously.^7^, ^39^ cDNA specifically from DCT cells was generated by using transgenic parvalbumin‐eGFP mice, as described previously.^40^ The cDNA was diluted 1:10 and used for qPCR using the iQ SYBR Green Supermix (Bio‐Rad, 1708887) according to manufacturer's instructions. The primers used were 5′‐ACGTCCCTGATCATTGGCTT‐3′ for *Slc41a3‐Iso 1* and 5′‐GTCGCCATACCTATCCTGCT‐3′ for *Slc41a3‐Iso 2*, with the reverse primer 5′‐CCAGCGTCATTTCCAGGTTT‐3′ used for both. *Gapdh* expression was used for normalization using primers 5′‐TAACATCAAATGGGGTGAGG‐3′ and 5′‐GGTTCACACCCATCACAAAC‐3′. The Livak (2^−ΔΔCt^) method was used to calculate relative expression.

### 4.2 Plasmids

pCIneo‐IRES‐GFP plasmids encoding HA‐tagged mouse SLC41A3‐Iso 1 and ‐Iso 2 (NM_027868.2 and NM_001037493.2) and human SLC41A3 isoform 1 and 4 (hSLC41A3‐Iso 1 and ‐Iso 4, NM_001008485.2 and NM_001008487.2) were constructed by VectorBuilder (https://en.vectorbuilder.com). Using these constructs as template, FLAG‐tagged SLC41A3‐Iso 1 and hSLC41A3‐Iso 4 constructs were generated for co‐transfection experiments. For immunofluorescence experiments, the GFP sequence was cut out of the vector using EcoRI and NotI restriction enzymes (New England Biolabs, R3101 and R3189). The vector was then ligated using annealed oligos with appropriately designed overhangs and T4 DNA ligase (New England Biolabs, M0202). The p.D204A variant in SLC41A3‐Iso 2 and hSLC41A3‐Iso 4 was generated using Q5 Site‐Directed Mutagenesis (New England BioLabs, E0554) following manufacturer's instructions. Commercially available plasmids encoding Firefly luciferase (Promega, E1751) and Renilla luciferase (Promega, E2261) were used for the luciferase assay. The 477 bp *Slc41a3‐Iso 2* cis‐regulatory element (CRE), the 1000 bp promoter, the 2837 bp promoter + region until CRE or the 3314 bp promoter + region until CRE + CRE were cloned in front of the Firefly luciferase. The required sequences were obtained from a Bacterial Artificial Chromosome clone (Source BioScience).

### 4.3 Cell culture and transfection

Human Embryonic Kidney 293 (HEK293) and HeLa cells were cultured in DMEM (Gibco 42 430–035) supplemented with 10% (v/v) fetal bovine serum, 1 mmol/L sodium pyruvate, and 0.1 mmol/L non‐essential amino acids (Westburg, CA NEAA‐B) at 37°C, 5% (v/v) CO_2_. HEK293 cells deficient in *CNNM3* and *CNNM4* (a kind gift from professor Loren W. Runnels) have been described previously^18^ and were cultured in the same conditions on poly‐L‐lysine‐coated surfaces (PLL, Sigma‐Aldrich, P2636). HAP1 WT and TRPM7 knock‐out (KO) cells were a kind gift from dr. Vladimir Chubanov^19^ and were cultured in IMDM (Gibco, 21 980–032) containing 10% (v/v) fetal bovine serum and 10 mmol/L MgCl_2_. All transfections were performed using Lipofectamine 2000 (Invitrogen, 11 668–019) using a DNA:Lipofectamine ratio of 1:2.

### 4.4 ^25^Mg^2^ ^+^ transport assays

HEK293 or HAP1 cells were seeded in 12‐well plates coated with poly‐L‐lysine and transfected with the respective mock or HA‐SLC41A3 isoform constructs. For uptake assays, cells were washed 48 h post‐transfection with transport buffer (125 mM NaCl, 5 mM KCl, 0.5 mM CaCl_2_, 0.5 mM Na_2_HPO_4_, 0.5 mM Na_2_SO_4_, 15 mM HEPES, set to pH 7.5 with NaOH) without Mg^2+^ and then exposed to transport buffer containing 1 mmol/L ^25^MgO (CortecNet, Voisins‐Le‐Bretonneux, France) for 15 minutes. Cells were then washed three times with ice‐cold PBS, lysed in HNO_3_ at 65°C for 15 minutes and analyzed with inductively coupled plasma mass spectrometry. For extrusion assays, cells were kept in their maintenance culture conditions up until 24‐h post‐transfection in PLL‐coated 12‐well plates. Subsequently, cells were loaded with ^25^Mg^2+^ by exposing the cells to Mg^2+^‐free medium containing 1 mmol/L ^25^MgO (first for 24 h, then for 48 h when 24 h resulted in unequal loading). The ^25^MgO was first dissolved in HCl and then further diluted in water. 48 h post‐transfection, cells were washed three times with transport buffer containing 1 mmol/L MgCl_2_, followed by an incubation using 0.2 mL transport buffer containing 0.5 mmol/L MgCl_2_ for 15 minutes. After collecting the buffer and adding 0.8 mL HNO_3_, the samples were processed in the same way as the uptake samples. To test for Na^+^‐dependent ^25^Mg^2+^ extrusion, the 125 mmol/L NaCl in the transport buffer was replaced with *N*‐Methyl‐D‐Glucamine (NMDG; Sigma‐Aldrich, M2004) or choline chloride (Sigma‐Aldrich, C7527) both during washing and extrusion. T = 0 timepoints, in which cells were exposed to transport buffer which was immediately removed, were included to determine basal levels of ^25^Mg^2+^, which were used for background subtraction for each experiment.

### 4.5 Immunocytochemistry

HeLa cells were seeded on PLL‐coated 12 mm coverslips and (co‐)transfected with 200 ng of the HA/FLAG‐SLC41A3 isoform constructs. 40 h post‐transfection, coverslips were washed twice with PBS and fixed with 4% formaldehyde (Thermo Scientific, 28 906) in PBS for 10 minutes at room temperature. After two washes with PBS, fixed cells were permeabilized in PBS with 0.3% (v/v) Triton X‐100 and 0.1% (w/v) BSA for 10 minutes and quenched in PBS with 50 mmol/L NH_4_Cl for 10 minutes. After washing twice with PBS, goat serum dilution buffer (GSDB; PBS with 16% (v/v) goat serum and 0.3% (v/v) Triton X‐100) was used for blocking for 30 minutes. Coverslips were incubated with primary antibodies (Table S1) diluted in GSDB overnight at 4°C. After three washes, secondary antibodies (Table S1) and 300 nM DAPI (Life Technologies, D1306) diluted in GSDB were added. Coverslips were mounted using Fluoromount‐G (SouthernBiotech, 0100–01). For mitochondrial stainings, coverslips were washed once with PBS and incubated in the dark at 37°C with 100 nM MitoTracker CMXRos (Thermo Scientific, M7512) in serum‐free medium for 45 minutes before the fixation.

### 4.6 Cell surface biotinylation

HEK293 cells were seeded in PLL‐coated 6‐well plates and transfected with 1 μg empty vector, 1 μg pCIneo‐HA‐SLC41A3‐Iso 2 or 20 ng pCIneo‐HA‐SLC41A3‐Iso 1 + 980 ng empty vector per well, to correct for the higher expression of SLC41A3‐Iso 1 compared to ‐Iso 2. 48 h after transfection, cells were transferred to a cold room (4°C), and washed twice with cold PBS‐CM (PBS with 0.5 mmol/L CaCl_2_ and 1 mmol/L MgCl_2_, set to pH 8.0 with NaOH). The cells were then incubated in PBS‐CM with 0.5 mg/mL Sulfo‐NHS‐LC–LC‐Biotin (Thermo Scientific, 21 338) for 30 minutes. For cells transfected with SLC41A3‐Iso 1 and ‐Iso 2, wells without biotin were included as negative controls. Cells were then washed twice in PBS‐CM with 0.1% (w/v) BSA and twice in PBS and subsequently lyzed in lysis buffer (1% (v/v) Triton X‐100, 50 mmol/L Tris–HCl pH 7.5, 1 mmol/L EDTA, 1 mmol/L EGTA, 10 mmol/L sodium glycerophosphate, 50 mmol/L NaF, 10 mmol/L sodium pyrophosphate, 270 mmol/L sucrose, 150 mmol/L NaCl) containing freshly‐added protease and phosphatase inhibitors (1 mmol/L phenylmethylsulfonyl fluoride, 5 μg/mL leupeptin hemisulfate, 1 μg/mL pepstatin A, 1 μg/mL aprotonin, 1 mmol/L sodium orthovanadate). Equal amounts of proteins were added to 20 μL NeutrAvidin Agarose Resin (Thermo Scientific, 29 201) and 1 μg/μl input samples were prepared from the same samples to check expression. The Resin samples were incubated overnight while rotating at 4°C. The samples were then washed three times with lysis buffer and eluted with 40 μL 2× Laemmli‐dithiothreitol buffer (4% (w/v) SDS, 0.02% (w/v) bromophenol blue, 12% (v/v) glycerol, 120 mM Tris–HCl pH 6.8, 200 mM dithiothreitol) at 37°C for 30 minutes.

### 4.7 Co‐immunoprecipitation

HEK293 cells were seeded in 100 mm Petri dishes and co‐transfected with a total of 5 μg of constructs encoding HA‐ or FLAG‐tagged SLC41A3 isoforms and GFP‐SLC41A1. 40 h post‐transfection, cells were harvested in the earlier described lysis buffer freshly supplemented with the protease and phosphatase inhibitors. After taking 50 μg of protein for the input sample, equal amounts of protein were incubated with anti‐FLAG (Sigma‐Aldrich, A2220) agarose beads for 2 h, rotating at 4°C. Beads were then washed three times with lysis buffer and proteins were eluted with 2× Laemmli‐dithiothreitol buffer.

### 4.8 Western blotting

Laemmli‐dithiothreitol samples were loaded on 10% SDS‐PAGE gels for gel electrophoresis. Proteins were then transferred to polyvinylidene difluoride membranes for 90 minutes at 100 V. Membranes were blocked with 5% (w/v) milk in TBS with 0.1% (v/v) Tween (TBS‐T) and incubated overnight with primary antibodies diluted in 1% (w/v) milk (Table S1) at 4°C. After four 10 minutes washes with TBS‐T, membranes were incubated with the secondary antibody (Table S1) for 1 h at room temperature. After three washes with TBS‐T and one with TBS, the signal was visualized with (Fisher Scientific, 34 578/ 34 095) chemiluminescent substrate.

### 4.9 Luciferase reporter assays

HeLa cells were seeded in 12‐well plates and co‐transfected with 700 ng of the different Firefly luciferase constructs and 20 ng of the Renilla luciferase construct used for normalization. 48 h post‐transfection, luciferase activity was determined using the Dual‐Luciferase Reporter Assay System (Promega, E1910) according to manufacturer's instructions. Measurements were performed on the Victor X3 plate reader (PerkinElmer).

### 4.10 In silico analyses

The distinct N‐termini of NP_082144.2, NP_001032570.1, NP_001008485.2 and NP_001008487.1 were aligned using MUSCLE^16^ and submitted to SignalP 6.0 to identify potential signal peptides.^41^ JASPAR 2020 was used to predict transcription factor binding sites in the identified regions upstream of the mouse *Slc41a3 isoform 2* and human *SLC41A3 isoform 4* transcription start sites, using a motif score cut‐off value of ≥400 (on a scale from 0 to 1000 where 0 corresponds to a *p*‐value of 1 and 1000 to 10^−10^).^42^

### 4.11 Statistical analysis

All data are represented as mean ± standard error of the mean (SEM), or ± standard deviation (SD) in the case of the colocalization data. An unpaired *t*‐test (DCT RNA, cell surface biotinylation data), one‐way ANOVA with Dunnett's test (24‐ and 48‐h loading experiments), or Welch's ANOVA with Dunnett's T3 test (luciferase assay) were used to compare group means. One‐Sample *t*‐tests were employed when means were compared to a set control value of 100%. *p*‐values of <0.05 were considered significant. However, for the one‐sample *t*‐tests a threshold of <0.025 was used to correct for the multiple testing of two isoforms.

## AUTHOR CONTRIBUTIONS

**Gijs A. C. Franken:** Conceptualization; investigation; visualization; formal analysis; writing – review and editing; writing – original draft. **Willem Bosman:** Writing – original draft; investigation; conceptualization; visualization; writing – review and editing; formal analysis. **Hyun Jun Jung:** Data curation; software; investigation. **Caro Bos:** Investigation; resources. **Femke Latta:** Investigation; resources. **Mark Knepper:** Conceptualization; software; writing – review and editing. **Joost G. J. Hoenderop:** Project administration; supervision; funding acquisition; writing – review and editing. **Jeroen H. F. de Baaij:** Project administration; supervision; funding acquisition; writing – review and editing.

## FUNDING INFORMATION

This work was supported by the IMAGEN (IMplementation of Advancements in GENetic Kidney Disease) project which is co‐funded by the PPP Allowance made available by Health∼Holland, Top Sector Life Sciences & Health, to stimulate public–private partnerships (LSHM20009) and the Dutch Kidney Foundation (20OP + 018). In addition, this work was funded by the European Research Council grant 101 040 682 (IN‐THE‐KIDNEY) and Netherlands Organization for Scientific Research grants Rubicon 452 022 311, OCENW.M.21.022 and VIDI 09150172110040 (IMAGE‐THE‐KIDNEY). Hyun Jun Jung was financially supported by the Edward S. Kraus Scholar Award, Johns Hopkins University School of Medicine Division of Nephrology.

## Supporting information


Data S1:

## Data Availability

The data that support the findings of this study are openly available in Gene Expression omnibus (GEO) at https://www.ncbi.nlm.nih.gov/geo/query/acc.cgi?acc=GSE150338, reference numbers GSE150338, GSE157079, and GSE200547.
