# Supplementary material for: A distal convoluted tubule‐specific isoform of murine SLC41A3 extrudes magnesium
Source: Acta Physiol (Oxf). 2025 Feb 11;241(3):e70018. doi: 10.1111/apha.70018 (PMC11811817; doi:10.1111/apha.70018)
Supplement: Supplementary file 1 — Data S1: [file APHA-241-e70018-s001.docx]

**SUPPLEMENTARY MATERIAL**

**Table S1.** Antibodies

| **Target** | **Species** | **Dilution** | **Manufacturer** | **Catalogue number** |
| --- | --- | --- | --- | --- |
| ***Immunocytochemistry*** | | | | |
| HA | mouse | 1:200 | Cell Signaling Technology | 2367 |
| FLAG | mouse | 1:500 | Sigma-Aldrich | F1804 |
| FLAG | rabbit | 1:160 | Sigma-Aldrich | F7425 |
| PDI | mouse | 1:1000 | Invitrogen | MA3-019 |
| 58K | mouse | 1:200 | Sigma-Aldrich | G2404 |
| LAMP1 | rabbit | 1:200 | Cell Signaling Technology | 9091 |
| mouse IgG | goat | 1:300 | Invitrogen | A11005 |
| rabbit IgG | goat | 1:300 | Invitrogen | A11008 |
| ***Western blotting*** | | | | |
| HA | mouse | 1:5000 | Cell Signaling Technology | 2367 |
| FLAG | rabbit | 1:1000 | Sigma-Aldrich | F7425 |
| β-actin | mouse | 1:10000 | Sigma-Aldrich | A5441 |
| Vinculin | mouse | 1:1000 | Santa Cruz Biotechnology | sc-25336 |
| mouse IgG | sheep | 1:10000 | Jackson ImmunoResearch | 515-035-003 |
| rabbit IgG | sheep | 1:10000 | Sigma-Aldrich | A4914 |

**Table S2.** Transcription factors with motifs found in the cis regulatory elements of both mouse *Slc41a3-Iso 2* and human *SLC41A3-Iso 4* with their RNA expression levels in mouse DCT.

| **Transcription factor** | **Expression in mouse DCT (TPM)^22^** |
| --- | --- |
| *Esr2* | 0.015 |
| *Foxj3* | 15.345 |
| *Kf1* | 0.790 |
| *Rara* | 4.213 |
| *Znf740* | ND |

DCT, distal convoluted tubule; ND, not detected; TPM, transcripts per million.

Mouse Iso 1 MEGTEARQRRLEGCGRLKELGPLPSHDA-GRLPKASEEGHLAVSESQLVDAKSLEAPPGR

Mouse Iso 2 ---MVVTQLSLEFRFQGKKLRGFSCELT-------------RSP--HGILP---------

Human Iso 1 MDGTETRQRRLDSCGKPGEL-GLPHPLSTGGLPVASEDGALRAPESQSVTPKPLETEPSR

Human Iso 4 ---MVVTQLNLEFCFQGKKLRGFSCELT-------------RSP--HGVLP---------

. * *: . :* :. : . : : .

Mouse Iso 1 ETSLIIGFQVVIPFLLAGVGLSWAGLLLNYFQHWPVFKDVKDLMTLVPPLVGLKGNLEMT

Mouse Iso 2 EPVLTTTCQVAIPILLSGLGMMTAGLVMNTVQHWPVFKDVKDLMTLVPPLVGLKGNLEMT

Human Iso 1 ETTWSIGLQVTVPFMFAGLGLSWAGMLLDYFQHWPVFVEVKDLLTLVPPLVGLKGNLEMT

Human Iso 4 ESFFTIMCQVVVPILLSGLCMMTAGLVMNTIQHWPVFVEVKDLLTLVPPLVGLKGNLEMT

*. **.:*::::*: : **:::: .****** :****:****************

Mouse Iso 1 LASRLSTSANTGQIDDRQERYKIISSNLAVVQVQATVVGLLAAVASLMLGTVSHEEFDWS

Mouse Iso 2 LASRLSTSANTGQIDDRQERYKIISSNLAVVQVQATVVGLLAAVASLMLGTVSHEEFDWS

Human Iso 1 LASRLSTAANTGQIDDPQEQHRVISSNLALIQVQATVVGLLAAVAALLLGVVSREEVDVA

Human Iso 4 LASRLSTAANTGQIDDPQEQHRVISSNLALIQVQATVVGLLAAVAALLLGVVSREEVDVA

*******:******** **.:.:******::**************:*:**.**.**.* :

Mouse Iso 1 KVALLCTSSVITAFLAALALGILMICIVIGARKFGVNPDNIATPIAASLGDLITLSILAL

Mouse Iso 2 KVALLCTSSVITAFLAALALGILMICIVIGARKFGVNPDNIATPIAASLGDLITLSILAL

Human Iso 1 KVELLCASSVLTAFLAAFALGVLMVCIVIGARKLGVNPDNIATPIAASLGDLITLSILAL

Human Iso 4 KVELLCASSVLTAFLAAFALGVLMVCIVIGARKLGVNPDNIATPIAASLGDLITLSILAL

** ***:***:******:***:**:********:**************************

Mouse Iso 1 MSSFFYSHKDTWYLTPLVCVGFLALTPLWLFIAKQNPPIMKILKYGWFPIILAMIISSFG

Mouse Iso 2 MSSFFYSHKDTWYLTPLVCVGFLALTPLWLFIAKQNPPIMKILKYGWFPIILAMIISSFG

Human Iso 1 VSSFFYRHKDSRYLTPLVCLSFAALTPVWVLIAKQSPPIVKILKFGWFPIILAMVISSFG

Human Iso 4 VSSFFYRHKDSRYLTPLVCLSFAALTPVWVLIAKQSPPIVKILKFGWFPIILAMVISSFG

:***** ***:.*******:.* ****:*::****.***:****:*********:*****

Mouse Iso 1 GLILSKTISKHEFKGMAVLTPVMCGVGGNLVAIQTSRISTFLHMWSTPGVLPVQMKRFWP

Mouse Iso 2 GLILSKTISKHEFKGMAVLTPVMCGVGGNLVAIQTSRISTFLHMWSTPGVLPVQMKRFWP

Human Iso 1 GLILSKTVSKQQYKGMAIFTPVICGVGGNLVAIQTSRISTYLHMWSAPGVLPLQMKKFWP

Human Iso 4 GLILSKTVSKQQYKGMAIFTPVICGVGGNLVAIQTSRISTYLHMWSAPGVLPLQMKKFWP

*******:**:::****::***:*****************:*****:*****:***.***

Mouse Iso 1 NPCFIFCSSEINSVSARVLLFLVVPGHLIFFYLICLVEGQSVTNSKIFILLYLVAGVVQV

Mouse Iso 2 NPCFIFCSSEINSVSARVLLFLVVPGHLIFFYLICLVEGQSVTNSKIFILLYLVAGVVQV

Human Iso 1 NPCSTFCTSEINSMSARVLLLLVVPGHLIFFYIIYLVEGQSVINSQTFVVLYLLAGLIQV

Human Iso 4 NPCSTFCTSEINSMSARVLLLLVVPGHLIFFYIIYLVEGQSVINSQTFVVLYLLAGLIQV

*** **:*****:******:***********:* ******* **: *::***:**::**

Mouse Iso 1 VILLYLAEVTVRLTWHQALDPDNHCIPYLTGLGDLLGTSLLA------------------

Mouse Iso 2 VILLYLAEVTVRLTWHQALDPDNHCIPYLTGLGDLLGTSLLA------------------

Human Iso 1 TILLYLAEVMVRLTWHQALDPDNHCIPYLTGLGDLLGSSSVGHTAAVPRRCTASPGWGLI

Human Iso 4 TILLYLAEVMVRLTWHQALDPDNHCIPYLTGLGDLLGTGLLA------------------

.******** ***************************:. :.

Mouse Iso 1 ---LC----------FFLDWLLRGRANLQELVSELVSVPP

Mouse Iso 2 ---LC----------FFLDWLLRGRANLQELVSELVSVPP

Human Iso 1 QPFICTQHLIVSLLSFYFPFCLLAKTSI------------

Human Iso 4 ---LC----------FFTDWLLKSKAELGG-ISELASGPP

:* *: : * ..:.:

**Figure S1. Alignment analysis of different SLC41A3 isoforms using MUSCLE.**

**Figure S2. Supplementary data for ^25^Mg^2+^ uptake and extrusion experiments.** (**A-B**) Intracellular ^25^Mg^2+^ content relative to ^24^ and ^26^Mg^2+^ in HEK293 cells transfected with mouse SLC41A3 or mock after 24 (A) or 48 (B) hours of incubation with ^25^Mg^2+^ medium. 48-hour loaded cells were used for the extrusion experiments. Data points represent three independent experiments. Data are presented as mean ± SEM. *P<0.05, ^†^P<0.01 compared to mock after one-way ANOVA with Dunnett’s test. (**C-D**) Representative Western blot (C) and quantification (D) of mouse SLC41A3-Iso 1 and -Iso 2 protein expression during the ^25^Mg^2+^ uptake and extrusion experiments in HEK293 cells. Data points represent five independent samples. Data are presented as mean ± SD. ^‡^P<0.001 after a one-sample T-test. (**E**) Representative Western blot of mouse SLC41A3-Iso 1 and -Iso 2 protein expression during the ^25^Mg^2+^ uptake experiments in HAP1 WT and TRPM7 KO cells. The low SLC41A3-Iso 2 expression is indicated with arrowheads. (**F**) Representative Western blot of human SLC41A3-Iso 1 and -Iso 4 protein expression during the ^25^Mg^2+^ uptake and extrusion experiments in HEK293 cells.
